# A high-throughput screening system for SARS-CoV-2 entry inhibition, syncytia formation and cell toxicity

**DOI:** 10.1186/s12575-023-00214-1

**Published:** 2023-07-26

**Authors:** Shine Varghese Jancy, Santhik Subhasingh Lupitha, Aneesh Chandrasekharan, Shankara Narayanan Varadarajan, Shijulal Nelson-Sathi, Roshny Prasad, Sara Jones, Sreekumar Easwaran, Pramod Darvin, Aswathy Sivasailam, Thankayyan Retnabai Santhoshkumar

**Affiliations:** Cancer Research Program, Rajiv Gandhi Centre for Biotechnology, Poojappura, Thycaud P.O., Thiruvananthapuram, Kerala 695014 India

**Keywords:** Severe acute respiratory syndrome coronavirus 2 (SARS-CoV-2), VSV-eGFP-SARS-CoV-2, Human angiotensin-converting enzyme 2, Biosafety level 2 (BSL-2), High-throughput drug screening, Reporter assay system, Syncytia, Molecular simulation

## Abstract

**Background:**

The entry of severe acute respiratory syndrome coronavirus 2 (SARS-CoV-2) into the host cell is mediated through the binding of the SARS-CoV-2 Spike protein via the receptor binding domain (RBD) to human angiotensin-converting enzyme 2 (hACE2). Identifying compounds that inhibit Spike-ACE2 binding would be a promising and safe antiviral approach against COVID-19.

**Methods:**

In this study, we used a BSL-2 compatible replication-competent vesicular stomatitis virus (VSV) expressing Spike protein of SARS-CoV-2 with eGFP reporter system (VSV-eGFP-SARS-CoV-2) in a recombinant permissive cell system for high-throughput screening of viral entry blockers. The SARS-CoV-2 permissive reporter system encompasses cells that stably express hACE2-tagged cerulean and H2B tagged with mCherry, as a marker of nuclear condensation, which also enables imaging of fused cells among infected EGFP positive cells and could provide real-time information on syncytia formation.

**Results:**

A limited high-throughput screening identified six natural products that markedly inhibited VSV-eGFP-SARS-CoV-2 with minimum toxicity. Further studies of Spike-S1 binding using the permissive cells showed Scillaren A and 17-Aminodemethoxygeldanamycin could inhibit S1 binding to ACE2 among the six leads. A real-time imaging revealed delayed inhibition of syncytia by Scillaren A, Proscillaridin, Acetoxycycloheximide and complete inhibition by Didemnin B indicating that the assay is a reliable platform for any image-based drug screening.

**Conclusion:**

A BSL-2 compatible assay system that is equivalent to the infectious SARS-CoV-2 is a promising tool for high-throughput screening of large compound libraries for viral entry inhibitors against SARS-CoV-2 along with toxicity and effects on syncytia. Studies using clinical isolates of SARS-CoV-2 are warranted to confirm the antiviral potency of the leads and the utility of the screening system.

**Supplementary Information:**

The online version contains supplementary material available at 10.1186/s12575-023-00214-1.

## Introduction

Worldwide, the coronavirus disease (COVID-19) has infected over 636 million people, and 6.6 million deaths have been reported [1]. The development of COVID-19 vaccines [2], neutralizing monoclonal antibodies [3], and antiviral therapies have played a crucial role in managing the pandemic. Recently, the Food and Drug Administration (FDA) approved the emergency use of two antiviral drugs, nirmatrelvir and molnupiravir, for non-hospitalized high-risk COVID-19 patients [4, 5]. Among the different stages of the viral life cycle, viral entry into the host is one of the most attractive therapeutic targets for drug discovery. Identifying a molecule that could modify or inhibit the binding of the Spike protein with the hACE2 receptor could be a promising therapeutic strategy against SARS-CoV-2 entry. Current vaccine development and antiviral therapy target this entry mechanism because it initiates the infection and these protein targets are accessible from the extracellular space [6, 7]. The global outbreak of multiple variants and mutations on the Spike protein causes viral escape from neutralizing antibodies, triggering the pandemic [8]. The continuing emergence of variants of concern and antibody escape mutants suggests the need for good viral entry blockers and better screening methods.

A large number of live virus assays, cell-free assays, and pseudoviral formats have been described for this purpose. High-throughput screening of compound libraries and repurposing have been reported with molecules that target viral entry and replication [9]. A screening of approved drugs in an entry-blocking assay identified seven drugs that could inhibit SARS-CoV-2 entry [10]. Despite intensive screening and drug discovery efforts, no compounds with SARS-CoV-2 entry inhibition [11] have reached the clinical setting. SARS-CoV-2 research requires a biosafety level 3 (BSL-3) facility, limiting research to a few laboratories worldwide. In this situation, most research laboratories use BSL-2-compatible pseudotyped viruses. Such pseudotyped viruses, supplemented by the expression of SARS-CoV-2 Spike protein, could enable the identification of viral entry inhibitors or neutralizers. In pseudotyped virion assays, glycoprotein expression is repeatedly performed by plasmid transfection, which needs optimization to reduce variation. Moreover, the reporter assay of pseudovirion relies on relative infectivity without correlation to infectious titre [12]. The Spike protein could also promote cell-to-cell fusion with ACE2-expressing cells, and this syncytia formation is associated with the severity of SARS-CoV-2 [13]. To identify antivirals that modulate cell fusion and syncytia formation, multiple methods have been described, including live virus or cells expressing Spike and ACE2 and a split gene reporter system [14]. Most of these approaches are time-consuming, cumbersome, and require multiple transfections.

The lack of a cell-based assay for testing the inhibition of viral entry and syncytia formation, along with host cell toxicity, is a significant concern in large-scale screening. In the present study, we utilized a BSL-2 compatible infectious clone of vesicular stomatitis virus (VSV) that encodes the SARS-CoV-2 S protein in place of the native envelope glycoprotein (G) developed by Whelan's lab [12]. This autonomously replication-competent virus, bearing the Spike protein with eGFP as a reporter of infection (VSV-eGFP-SARS-CoV-2), is easy to expand in permissive cells that express both ACE2/TMPRSS2 and performs equivalently to clinical isolates of SARS-CoV-2. Utilizing this clone, we have developed an assay system that can simultaneously detect SARS-CoV-2 viral entry inhibition, syncytia formation, and toxicity of the tested molecule. To facilitate single-cell identification for high-throughput application and cell death detection based on the condensed and fragmented nuclei, the cells were stably expressed with H2B mCherry. The assay system described here, with fluorescently tagged H2B mCherry and hACE2 Cerulean, could facilitate evaluating the Spike-ACE2 mediated cell-to-cell syncytia formation that is easy to implement in microscopy in real-time mode.

## Materials and methods

### Development of SiHa ACE2 Cerulean H2B mCherry stable cell line

The full-length human ACE2 coding region with C-terminal Cerulean was inserted into the mammalian expression vector under the control of the CMV promoter to generate the pRP-Puro-CMV-hACE2 Cerulean vector (pRP[Exp]-Puro-CMV > hACE2[NM_021804.3](ns):Cerulean), which was custom-synthesized from Vector Builder. The human H2B-mCherry expression vector pRP-Neo-CMV-hH2BC3 [NM_021062.3] mCherry was cloned into a mammalian expression vector with a neomycin resistance cassette through custom synthesis from Vector Builder.

SiHa is a cervical cancer cell line isolated from a patient with squamous cell carcinoma. This cell line is a suitable transfection host and also expresses transmembrane serine protease 2 (TMPRSS2). SiHa cells were first transfected with ACE2 (pRP-Puro-CMV-hACE2 Cerulean) using FuGENE HD transfection reagent and were maintained in selection media containing Puromycin for two weeks. The Cerulean-expressing cells were sorted using FACS based on green fluorescence intensity excited by the 488 nm laser line (BD FACSAria III). The sorted cells were grown in 6-well plates at low density. Multiple clones expressing varying levels of ACE2 were expanded and used for further transfection. The expression of ACE2 and TMPRSS2 were confirmed using western blot technique using rabbit anti h ACE2 antibody (SAB3500346, Sigma Aldrich) and rabbit anti TMPRSS2 antibody (Cat No. HPA035787, Sigma Aldrich) as per the standard protocol.

Clones of stably expressing ACE2 Cerulean were further transfected with the plasmid encoding H2B-mCherry using FuGENE HD transfection reagent and were maintained in selection media containing G-418 for two weeks. Cells were further sorted based on red fluorescence intensity excited by the 562 nm laser to get cells expressing both transgenes. The cells were grown in 6-well plates at low density. Multiple clones expressing varying levels of H2B-mCherry and ACE2 Cerulean were identified under fluorescent microscopy, and better clones expressing both ACE2 Cerulean and H2B-mCherry were expanded for the present study.

### Infectious VSV clone pseudotyped with SARS-CoV-2 for natural compound screening

The infectious clone of VSV pseudotyped with SARS-CoV-2 Spike protein expressing eGFP (VSV-eGFP-SARS-CoV-2) was obtained as a gift from Prof. Whelan. The infectious clone was amplified in HEK 293 T cells overexpressed with ACE2 and TMPRSS2. Briefly, HEK cells stably expressing hACE2 were transfected with TMPSS2 (pRP[Exp]-Hygro-CMV > Myc/hTMPRSS2[NM_005656.4) which was custom synthesised through Vector Builder and selected using Hygromycine. The double stable cells were used for viral stock preparation as described earlier [12]. Briefly, the virus was used to infect the target cells at a multiplicity of infection (MOI) of 0.3 for 1 h, after which the inoculum was replaced with fresh media containing 5% FBS. The cell supernatant was harvested upon visualization of the extensive cytopathic effect of GFP-positive cells. The cell supernatant was centrifuged for 5 min at 1,000 × g, and aliquots of the supernatant (up to 5 passages) were either stored at -80 °C or used immediately for experiments after calculating the Viral titre. Viral titre was calculated by visualizing the number of GFP-positive plaques using a fluorescent microscope 24 h post-infection (hpi).

### Validation of reporter assay system for pseudovirion infection

SiHa parental cells and SiHa ACE2 Cerulean H2B mCherry cells were cultured in 96-well glass bottom plates. After 24 h, the cells were exposed to the infectious clone of VSV-eGFP-SARS-CoV-2 (MOI-0.3) and incubated for 1 h in a humidified 37 °C CO_2_ incubator. After incubation, the cells were allowed to grow in fresh media and imaged at 24 hpi using a Nikon A1R confocal microscope. For imaging H2B mCherry, cells were excited using a 561 nm laser and emission was collected at 595/24 nm. For imaging ACE2 Cerulean, cells were excited using a 405 nm laser and emission was collected at 450/30 nm. To visualize EGFP, cells were excited using a 488 nm laser and emission was collected at 525/20 nm. The images were further analyzed using NIS element software.

### Validation of reporter assay system for syncytia formation

For real-time imaging of syncytia formation, SiHa ACE2 Cerulean H2B mCherry cells were grown in 96-well glass bottom plates and incubated with VSV-eGFP-SARS-CoV-2 for 1 h. The media was then replaced with fresh media, and time-lapse imaging was performed with 1-h intervals using Nikon A1R confocal microscope, 20 X objective with 2X zoom for 32 h. During live imaging, cells were maintained at 37 ℃ in 5% humidified CO2 throughout the experiments with the help of an onstage incubation chamber from Tokai Hit (Japan). To image ACE2 Cerulean, cells were excited using 405 laser and emission was collected at 450/30. The images of cerulean, mCherry and EGFP were further analysed using NIS element software. Syncytia formation was assessed by co-expression of EGFP and H2B mCherry within a single multinucleate fused ACE2 Cerulean membrane scored under microscopy.

### Validation of SARS-CoV-2 permissive reporter system for nuclear condensation and viral entry by microscopy

To validate the nuclear condensation and viral entry by widefield fluorescent microscopy, SiHa cells expressing both ACE2 cerulean and H2B mCherry, were exposed to cytotoxic Arsenic trioxide in varying concentrations. The clinically approved monoclonal antibody against Spike protein, Casirivimab (N7557B01, Ronapreve, Roche) was used as a positive control. For imaging H2B mCherry, cells were excited using 561 nm light from Lumencore LED light source and emission was collected at 570–620 nm using Nikon TiEclipse microscope aided with 40 × 0.9 NA objective. For eGFP, the cells were excited with 488 nm light and emission at 500–550 nm was collected. Cytotoxicity is scored from the H2B signal if the nuclei shows marked condensation or fragmentation with loss of homogeneous red fluorescence in comparison with untreated control.

### High-throughput suitability of pseudovirion assay system

The NCI natural products set IV-13160330 (https://dtp.cancer.gov/organization/dscb/obtaining/available_plates.html) containing 240 natural products were tested using a high-throughput screening system. We pre-incubated VSV-eGFP-SARS-CoV-2 (MOI- 0.3) with 10 µM concentration of natural products for 1 h and then treated SiHa ACE2 cerulean H2B mCherry cells, which were seeded in a 96-well optical bottom plate, for 1 h at 37 ℃. Following this, cells were incubated with fresh media at 37 ℃ for 12 h and images were captured using high-throughput BD Pathway 435 Bioimager (Becton Dickinson Biosciences, USA) with a dry 20X objective and NA 0.75. GFP and mCherry imaging was carried out using specific band pass filters and analyzed using Attovision software. The primary hits were further evaluated for a dose–response study up to a 1 µM concentration. We evaluated the reconfirmed positive hits at the different concentrations for IC-50 studies (see Table 1). Simultaneously, we captured microscopic imaging using a 4X objective employing GFP and mCherry filter sets on a Nikon Eclipse Ti microscope. The images were further analyzed and segmented using the Image J software for scoring infection area against a number of cells identified based on mCherry spot.

**Table 1:** High-throughput screening of NCI library of 240 natural products (Natural products set IV 13160330 https://dtp.cancer.gov/organization/dscb/obtaining/available_plates.htm)

Summary of High-throughput screening	
Total number of Natural products screened	240
Initial Screening Concentration of 240 Natural products	10 µM
Primary Hits (> 80% inhibition of infection)	24
Hit Rate (%)	10
Positive hits after reconfirmation of primary Hits with dose response up to 1 µM	6
Reconfirmation rate (%)	16.6
EC_50_ < 1 µM	6

### Testing of compounds that interfere with hACE2 or rVSV-G-GFP

To exclude compounds that affect viral entry through its direct activity on host h ACE2 or rVSV-G-GFP replication, additional experiments were performed. For testing the effect on host cell ACE2 protein, SiHa ACE2 cerulean-H2B mCherry cells were pre-incubated with the test compounds for 1 h. After 1 h of pre-treatment, media was replaced with VSV-eGFP-SARS-CoV-2 treatment for 1 h at 37 ℃. Then the cells were incubated with fresh media at 37 ℃ for 12 h and imaged using a 4X objective employing GFP and mCherry filter sets on a Nikon Eclipse Ti microscope. The images were further segmented and analyzed using the NIS Element software.

The recombinant VSVΔG*G-GFP virus procured from Kerafast (#EH1019-PM) was used to test compounds that affect rVSV-G-GFP replication. To obtain working stocks of VSV-G-GFP pseudotyped with G glycoprotein, HEK 293 T cells were transfected with the plasmid pCAGGS-G 24 h prior to infection with VSVΔG*G-GFP at 0.1 MOI. The supernatant is collected 24 h post-infection filtered using 0.45-micron filter and used immediately for rVSV-G-GFP infection studies or stored at -80 ℃.

The rVSV-G-GFP viral supernatant was pre-incubated with the natural compounds for 1 h at 37 ℃. The preincubated viral-supernatant-compound mixture was used for infecting the SiHa ACE2-Cerulean and H2B mCherry cells. The infection was visualized from the GFP fluorescence 12–16 h post-infection using Nikon inverted fluorescent microscope and was further analyzed using NIS elements software.

### Time-lapse imaging of selected natural products

Cells were grown in glass-bottomed 96-well plates for 24 h and were then treated with pre-incubated (1 h) lead compounds with VSV-eGFP-SARS-CoV-2 for 1 h, after which they were replaced with fresh media. The IC_50_ concentration of natural products was used to monitor syncytia formation. After 4 h of VSV-eGFP-SARS-CoV-2 infection time-lapse imaging was performed at 1-h intervals using a Nikon A1R confocal microscope with a 20X objective and 2 × zoom for 20 h. During live imaging, cells were maintained at 37 ℃ in 5% humidified CO2 throughout the experiments with the help of an onstage incubation chamber from Tokai Hit (Japan). For imaging H2B mCherry, cells were excited at 587 nm, and emission was collected at 610 nm. For ACE2 Cerulean, excited at 433 nm, emission was collected at 475 nm. To visualize EGFP, cells were excited at 488 nm, and emission was collected at 510 nm using specific bandpass filters. The images were further analyzed using NIS element software.

### ACE2– spike S1- binding study

HEK293T cell was transfected with S1-Orange Fluorescent Protein Spark (PCMV3-2019-N CoV-S1-OFP Spark (# VG40591-ACR, SinoBiotech), and multiples clones with variable expression levels was expanded. HEK293T cells expressing a high level of S1-OFP was developed employing flow sorting and colony selection. Supernatant from the stable HEK293T S1-OFP cell was used for the binding studies.

SiHa ACE2 Cerulean stable cells were seeded on a 96-well glass-bottom plate, after 24 h cells were fixed using 4% paraformaldehyde. Compounds and human monoclonal antibody against spike Casirivimab, Indevimab (N7557B01, N7558, Ronapreve, Roche) were incubated with Spike S1-OFP cell supernatant for 1 h. SiHa ACE2 cerulean cells grown on 96 well glass bottom plates were fixed and treated with S1-OFP with/without Compound mix for 15 min [15]. After 15 min, images were captured using Nikon A1R confocal microscope with a 20X objective. ACE2 Cerulean and Spike OFP Spark were imaged with excitation at 440 nm, and 560 nm laser lines, and emissions of 450 – 490 nm and 570 to 630 nm were collected in sequential mode.

### Statistical analysis

Statistical analysis was performed using GraphPad Prism version 9.1.1.

## Results

### Development of SARS-CoV-2 permissive stable cells

**Fig. 1:**
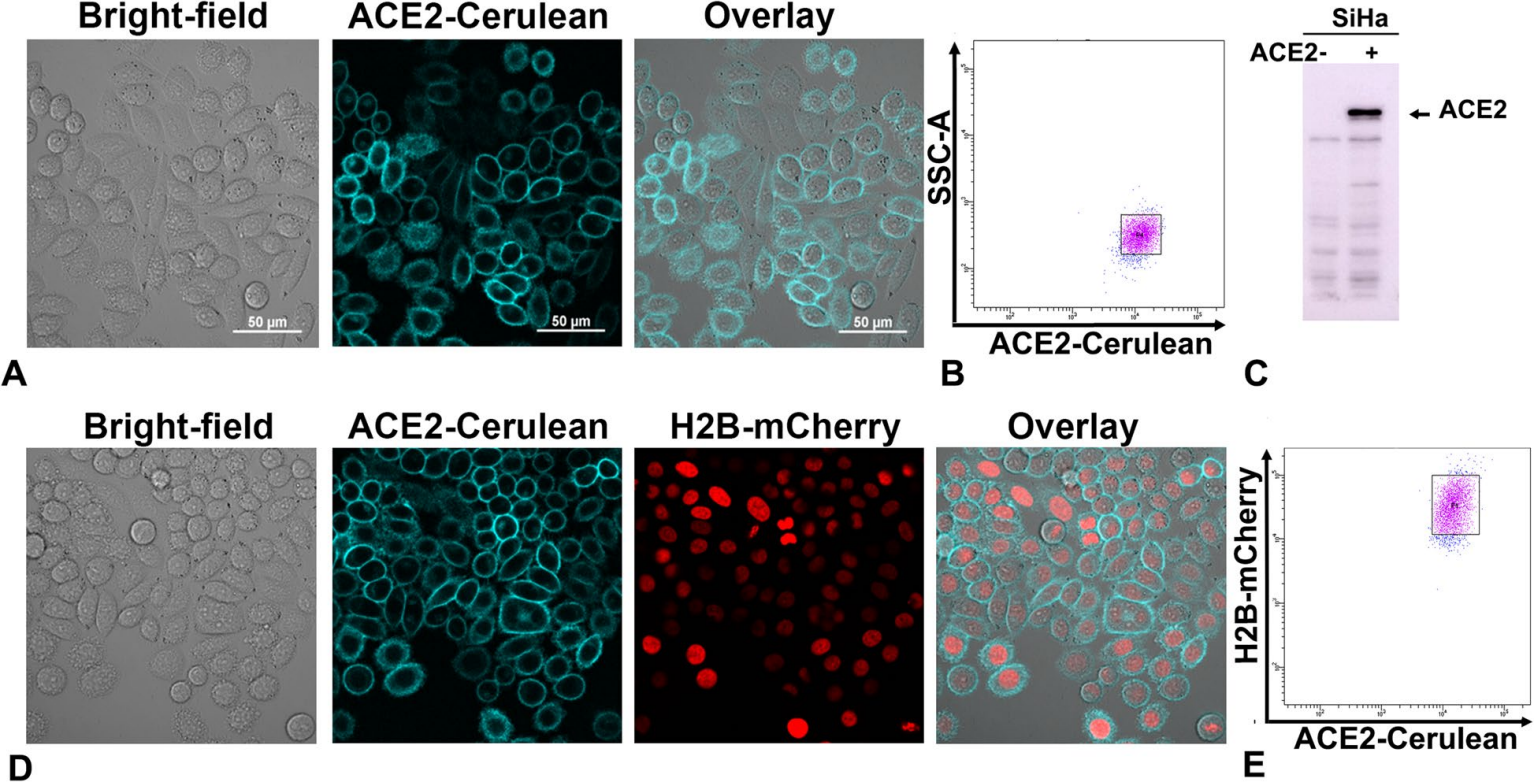
Development of SiHa ACE2 Cerulean H2B mCherry reporter system. **A** Microscopic images of SiHa ACE2 Cerulean stable cells. **B** Flow cytometry side scatter plot of SiHa ACE2 Cerulean cells, tight gate showed the selected population for sorting. **C** Western blot of overexpressed ACE2 protein in SiHa Cells. **D** Microscopic images of SiHa cells stably expressing ACE2 Cerulean and H2B mCherry. **E** Flow cytometry scatter plot of SiHa ACE2 Cerulean H2B mCherry cells

SARS-CoV-2 permissive stable cells were developed by overexpressing ACE2 protein in SiHa cells (Fig. 1A). To enable imaging applications along with an EGFP reporter (used as a pseudovirion infection reporter), ACE2 was expressed as a cerulean fusion protein, which was sorted using FACS (Fig. 1B and Supplementary Fig. 1). The overexpression of ACE2 was confirmed using immunoblot (Fig. 1C). SiHa ACE2-cerulean stable cells were further transfected with H2B mCherry and sorted using FACS (Fig. 1D-E) to enable high-throughput automated segmentation and toxicity evaluation of natural products simultaneously. SiHa cells express moderate TMPRSS2, which was confirmed by western blot and flow cytometry (Supplementary Fig. 2).

### Validation of SARS-CoV-2 permissive reporter system with pseudovirion infection

**Fig. 2:**
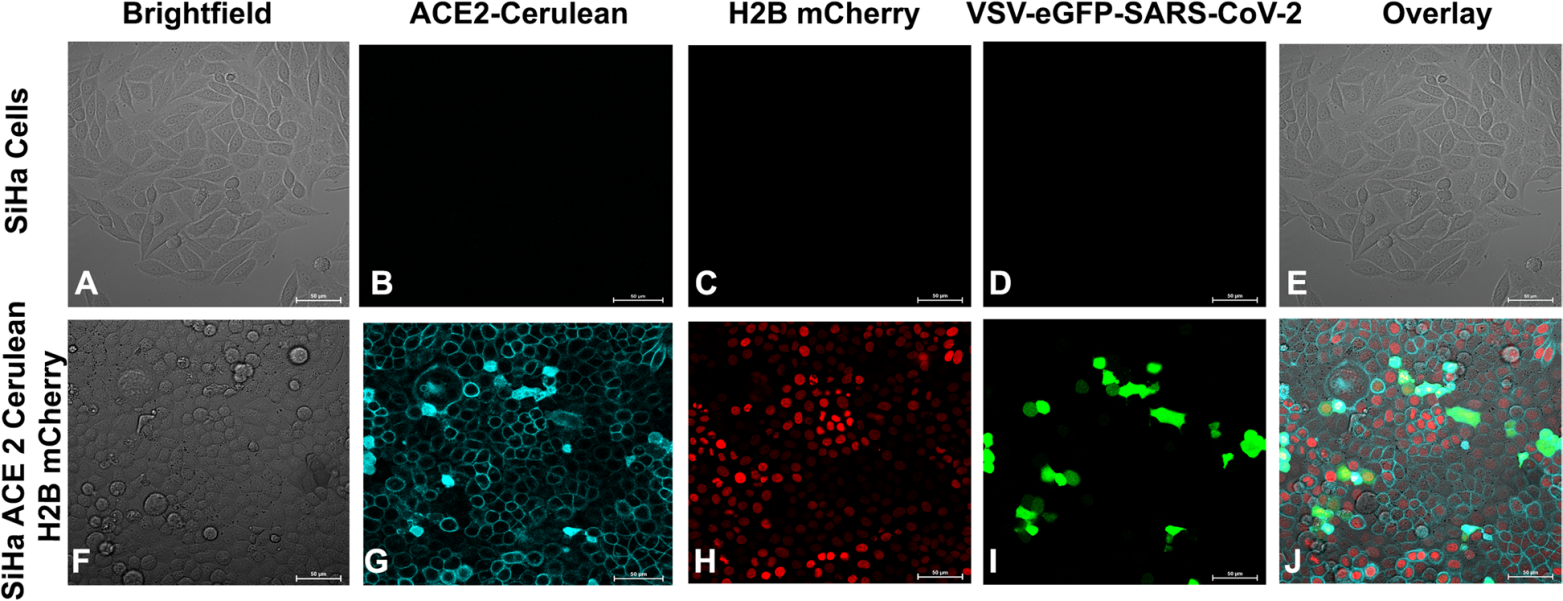
Validation of SiHa ACE2 Cerulean H2B mCherry cells using VSV-eGFP-SARS-CoV-2. **A**-**E** Microscopic images of SiHa cells infected with VSV-eGFP-SARS-CoV-2. **F**-**J** SiHa ACE2 Cerulean H2B mCherry cells infected with VSV-eGFP-SARS-CoV-2. **I** VSV-eGFP-SARS-CoV-2 infection was detected as eGFP plaques

Pseudovirion-VSV-eGFP-SARS-CoV-2 is an infectious clone of vesicular stomatitis virus (VSV) with GFP reporter developed by Prof. Whelan lab. The chimeric VSV displays the S protein in place of glycoprotein G, and resembles infectious SARS-CoV-2, so predominantly entry inhibitions are determined at early time point assays. SiHa ACE2 Cerulean H2B mCherry cells were incubated with pseudovirion-VSV-eGFP-SARS-CoV-2 for 1 h, and real-time imaging was performed, revealing GFP expression as an indication of pseudovirion infection initiated by 5 h (Supplementary Video 1). The observation showed that the developed reporter system is permissive to VSV-eGFP-SARS-CoV-2. In contrast, parental SiHa cells remained uninfected with VSV-eGFP-SARS-CoV-2 due to the absence of ACE2 cerulean (Fig. 2A-E). The results demonstrated the sensitivity and permissivity of VSV-eGFP-SARS-CoV-2 in SiHa ACE2 Cerulean cells for real-time imaging. Moreover, the H2B mCherry in the reporter system could facilitate segmentation and toxicity analysis (Fig. 2F-J). The developed assay system showed similar performance in wide-field and confocal imaging platforms.

### Validation of SARS-CoV-2 permissive reporter system for syncytia formation with VSV-eGFP-SARS-CoV-2 infection

**Fig. 3:**
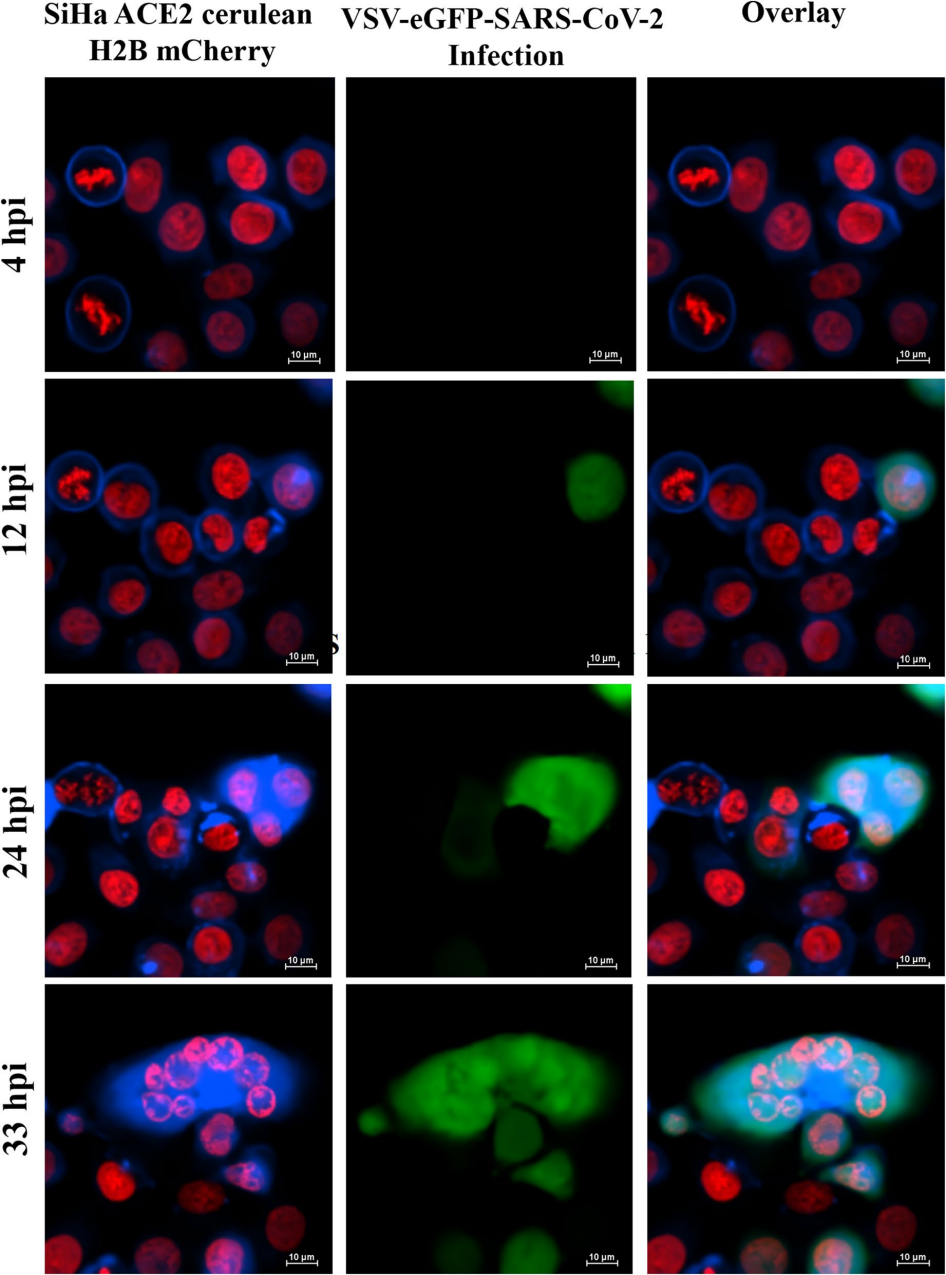
Validation for syncytia formation in SiHa ACE2 Cerulean H2B mCherry cells infected with VSV-eGFP-SARS-CoV-2. The time-lapse images showed syncytia formation at different time points up to 33 hpi

Time-lapse imaging of SiHa ACE2 Cerulean H2B mCherry cells, following 5 hpi of VSV-eGFP-SARS-CoV-2, demonstrated the initiation of infection through the expression of EGFP. Real-time monitoring of EGFP-positive cells revealed ACE2-Spike-dependent syncytia formation (Fig. 3 and Supplementary Video 2), characterized by membrane fusion of multiple cells using membranous ACE2-Cerulean and H2B mCherry nuclei. This confirmed the fusion activity from 12 hpi onwards. Steady-state imaging of cells 12 hpi for Cerulean and H2B mCherry enabled the quantification of multinucleate fused cells as a measure of syncytia formation. Overall, the results demonstrate that the reporter assay system, utilizing membranous expression of ACE2 Cerulean and nuclear expression of H2B mCherry, can effectively identify potential candidates that inhibit ACE2-Spike-dependent syncytia formation (Fig. 3).

### Validation of SARS CoV-2 permissive reporter system for inhibition of VSV-eGFP-SARS-CoV-2 infection and nuclear condensation

**Fig. 4:**
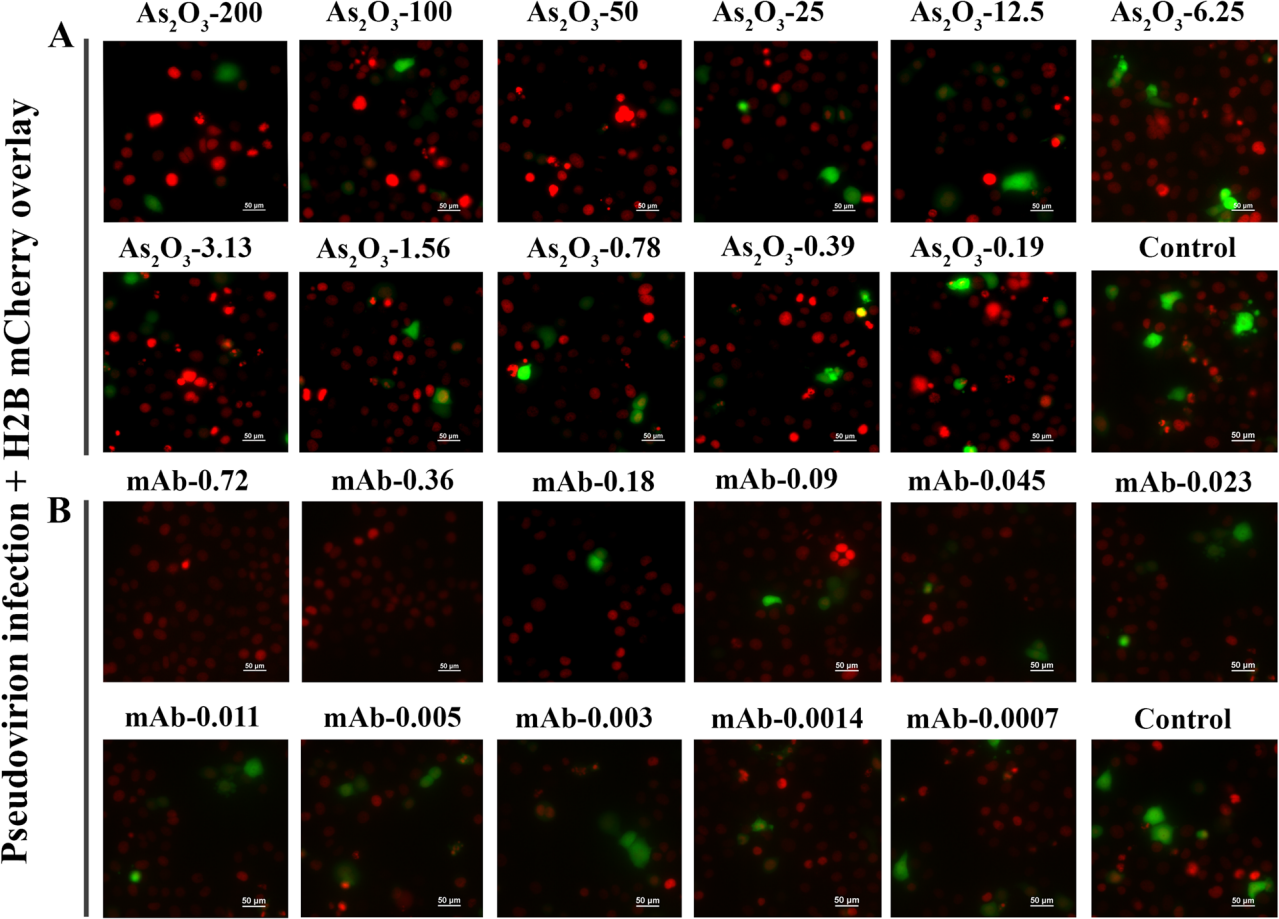
Validation of SiHa ACE2 Cerulean H2B mCherry cells using known cytotoxic compound Arsenic trioxide (As2O3—µM) anti-spike human monoclonal antibody Casirivimab (mAb—nM). Control—VSV-eGFP-SARS-CoV-2 infection without drug. The nuclear condensation is visible in Arsenic treatment

A SARS-CoV-2 permissive cell line has been validated using different MOI of pseudovirion (0.7 to 0.01) and known viral entry inhibitor Casirivimab monoclonal antibody against spike protein (Fig. 4). Casirivimab showed inhibition of VSV-eGFP-SARS-CoV-2 infection without any cellular toxicity in higher concentration, whereas, cellular toxicity was increased with antibody dilution (Supplementary Fig. 3 and Fig. 4) The known cytotoxic compound, arsenic trioxide, showed concentration-dependent nuclear condensation indicating cellular toxicity without inhibiting pseudovirion infection (Fig. 4).

### High-throughput suitability of pseudovirion assay system

**Fig. 5:**
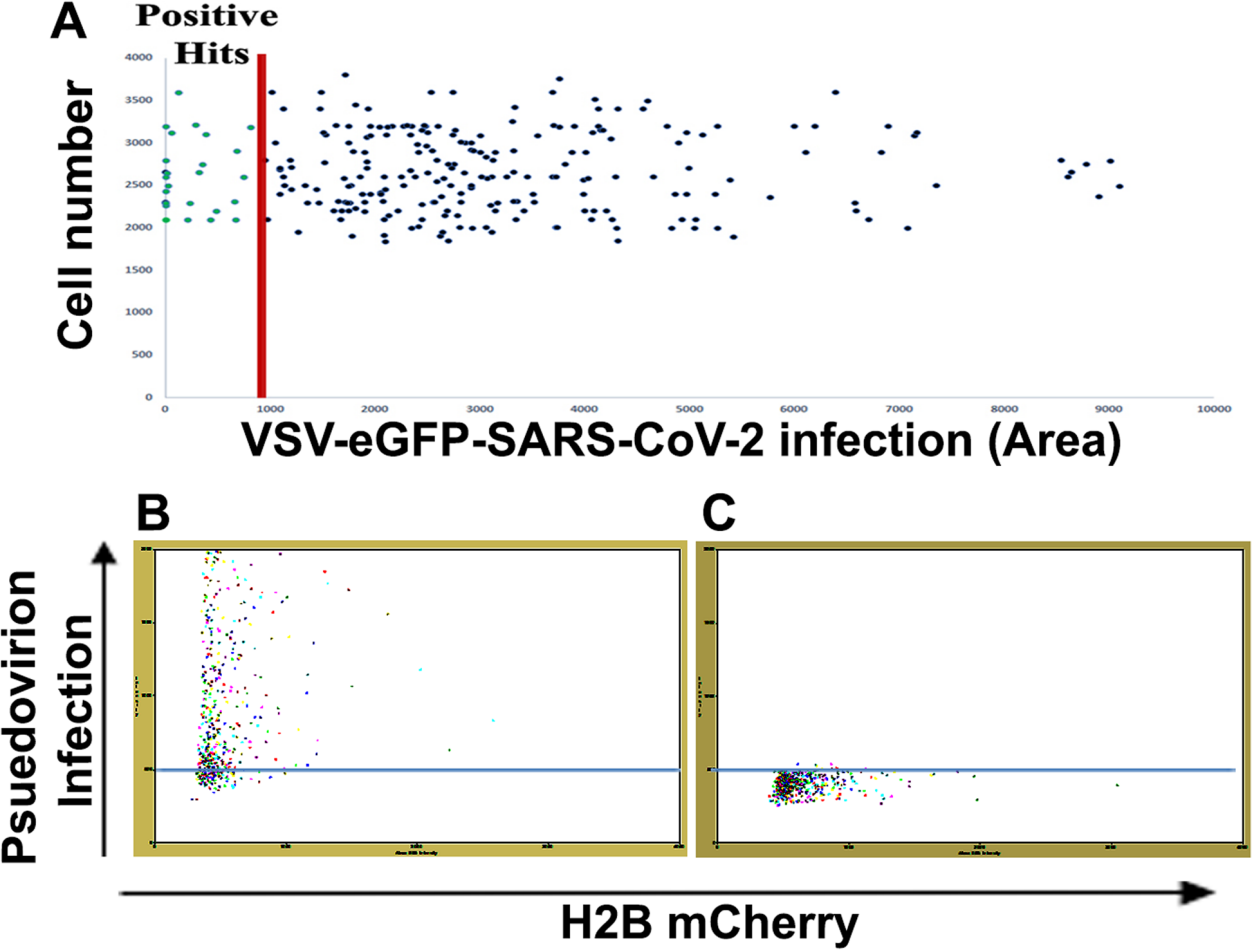
High-throughput screening of NCI library of 240 natural products using BD Pathway 435 Bioimager **A** Graph showing 24 positive hits (marked green) at an initial concentration of 10 µM (> 80% inhibition) Area (µm2), Cell number calculated by counting H2B mCherry and FITC expression area (Pseudovirion infection) using ImageJ, each dot in the graph represents each compound. **B** System-generated segmentation analysis for total count and area of objects of a sample with VSV-eGFP-SARS-CoV2 infection (AU), each dot represents each cell. **C** System-generated segmentation analysis for total count and area of objects of a sample without VSV-eGFP-SARS-CoV2 infection, each dot represents each cell. Data mean ± S.D n = 3 replicate experiments compared to DMSO control

**Fig. 6:**
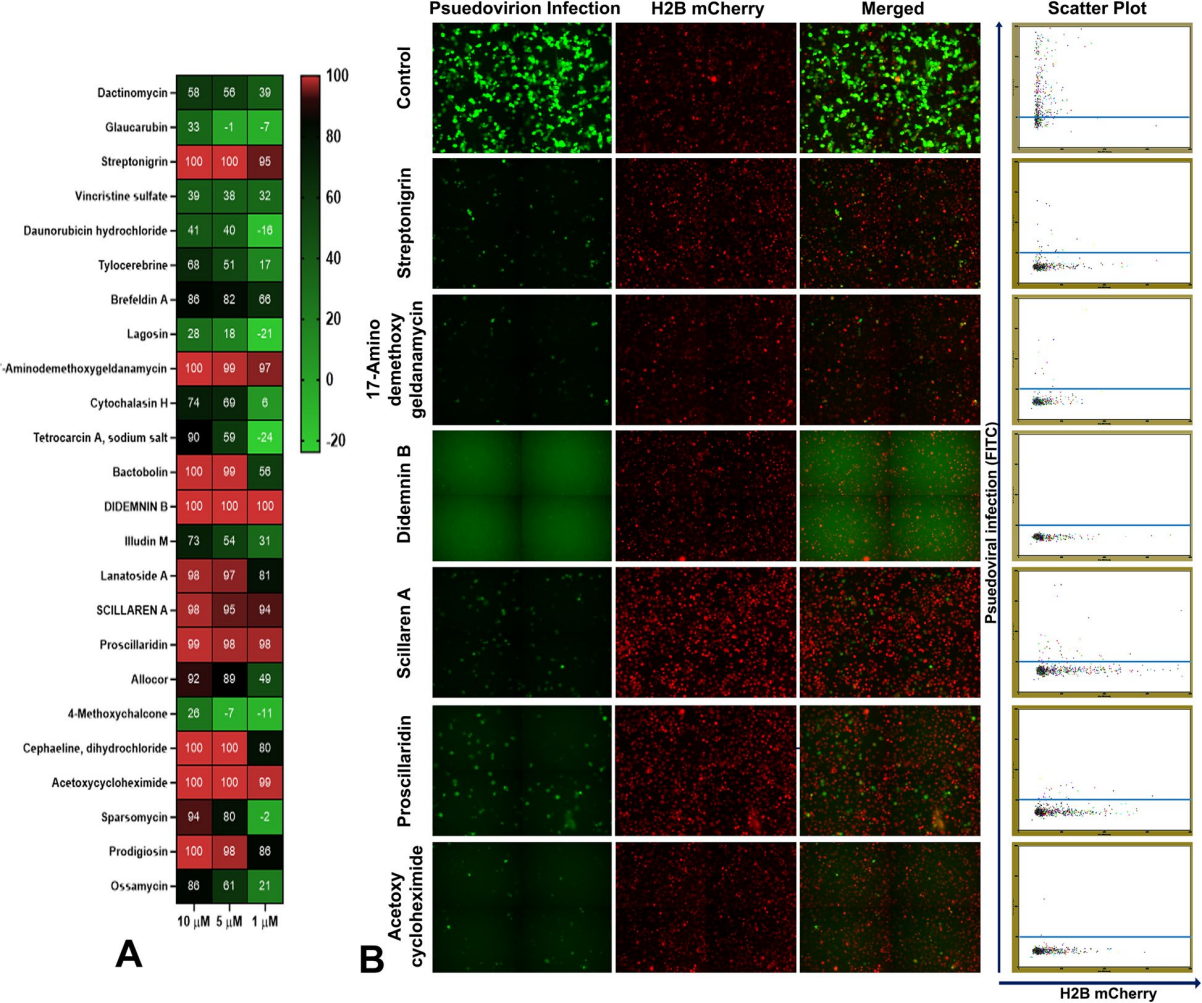
**A** Heatmap of dose response (10, 5, 1 μM) of 24 primary hits against VSV-eGFP-SARS-CoV2 infection (12 hpi), Six compounds showing inhibitory activity at 1 μM concentration (> 80% inhibition). **B** High-throughput analysis of six natural products 10 μM concentration using BD Pathway 435 Bioimager

**Fig. 7:**
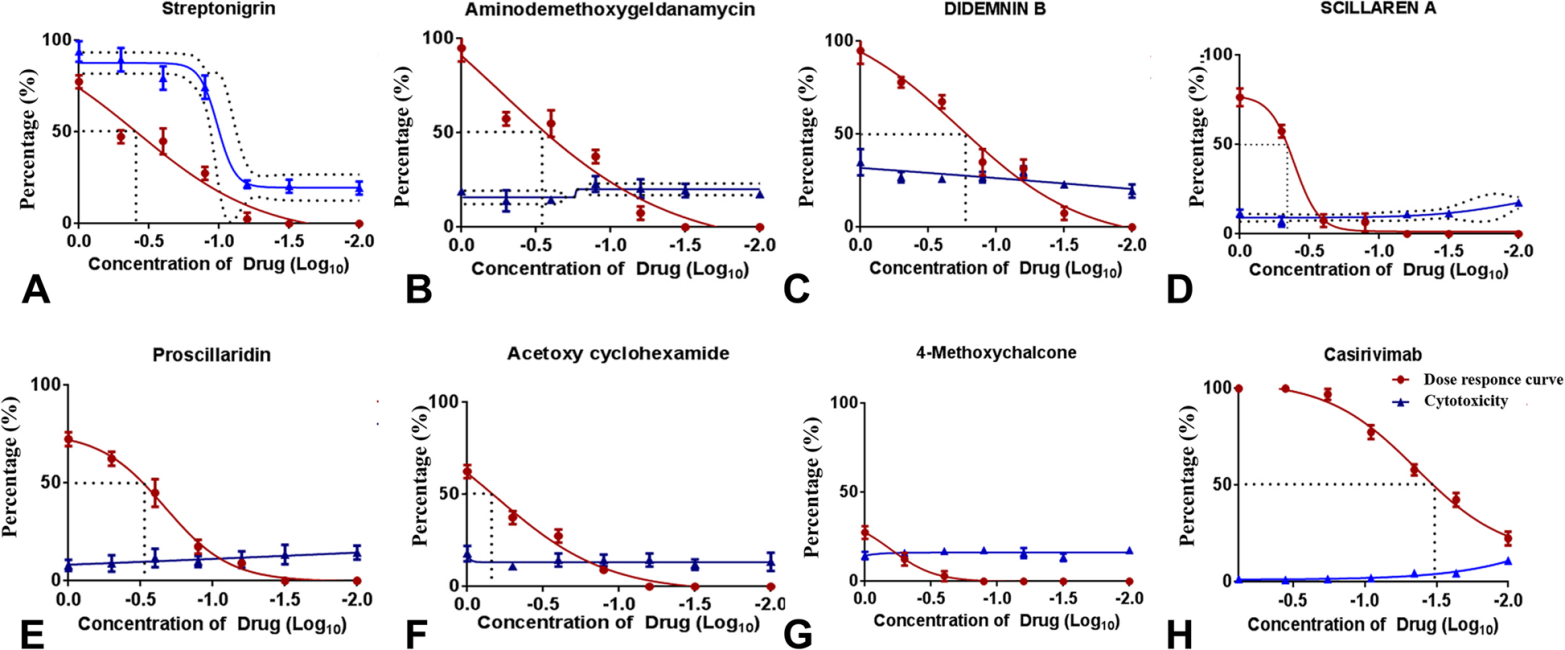
Dose–response curve and cytotoxicity of Six natural products against VSV-eGFP-SARS-CoV2 infection. IC-50 Values of six compounds **A** Streptonigrin (0.38 μM), **B** 17-Aminodemethoxygeldanamycin (0.56 μM), **C** Didemnin B (0.19 μM), **D** Scillaren A (0.41 μM), **E** Proscillaridin (0.22 μM), **F** Acetoxycyclohexamide (0.64 μM) **G **negative control 4-methoxychalcon **H** Antispike human monoclonal antibody Casirivimab (0.045 nM) and were estimated. Data mean ± S.D n = 3 replicate experiments compared to DMSO control, 12 hpi

In order to test the assay system for low-cost image-based screening applications, we have used conventional fluorescent microscopy using 4 × objective and software package image J for quantitation of infection. Since it is difficult to score cytotoxicity using 4 × images, H2B mCherry signal was used only for segmentation to identify the number of cells in a field against the infection area for each compound. The natural product libraries from NCI, consisting of 240 compounds, were tested using the system. The assay was conducted at an initial concentration of 10 µM and identified 24 positive hits with more than 80% inhibition of VSV-eGFP-SARS-CoV-2 infection (Fig. 5A). The high-throughput experimental design and quantitative analysis for viral entry inhibition using the assay system are detailed in Table 1 and Fig. 5B. The 24 positive hits were further tested for dose–response studies (10, 5 & 1 µM) with toxicity analysis. The segmentation analysis of the dose–response study revealed six natural products, Streptonigrin,17-Amino demethoxygeldanamycin, DidemninB, Scillaren A, Proscillaridin, and Acetoxycycloheximide, exhibited more than 50% inhibition at 1 µM concentration (Fig. 6 & Table 1). Further toxicity evaluation from H2B mCherry condensation has been quantified manually with and without VSV-eGFP-SARS-CoV-2. The results revealed that streptonigrin and Didemnin B exhibited toxicity at higher doses (Supplementary Fig. 4 and Fig. 7). However, streptonigrin and Didemnin B showed minimum toxicity at IC50 dose with and without VSV-eGFP-SARS-CoV-2 infection. The IC_50_ values of Streptonigrin (0.38 μM), 17-Aminodemethoxygeldanamycin (0.56 μM), Didemnin B (0.19 μM), Scillaren A (0.41 μM), Proscillaridin (0.22 μM), Acetoxycyclohexamide (0.64 μM) and known anti-spike antibody Casirivimab (0.045 nM) were calculated (Fig. 7). VSV-eGFP-SARS-CoV-2 infection also induced nuclear condensation up to 17%. Preincubation of natural products with SiHa ACE2 Cerulean H2B mCherry cells and further VSV-eGFP-SARS-CoV2 infection showed Didemnin B and 17-Aminodemethoxygeldanamycin having inhibitory activity of 1 μM concentration. Pre-treatment of Aminodemethoxygeldanamycin showed 50% inhibition, whereas along with VSV-eGFP-SARS-CoV-2 showed 97% inhibition (Supplementary Fig. 5).

### Screening of positive hit compounds with rVSV-G-GFP infection

**Fig. 8:**
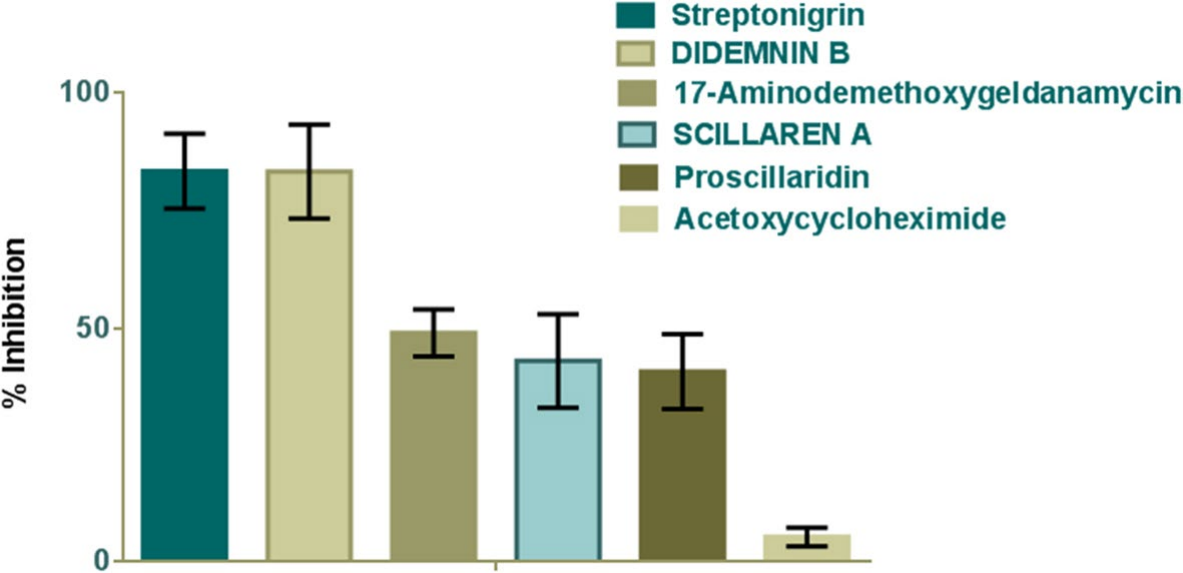
Screening with rVSV-G-GFP infection. Graph showing rVSV-G-GFP inhibition streptonigrin, Didemnin B treatment. 17-Aminodemethoxy geldanamycin, scillaren A, Proscillaridin, and Acetoxycyclohexamide showed moderate inhibition. Graph mean ± SD, n = 3

Screening of six positive hits with rVSV-G-GFP infection revealed that Streptonigrin, and Didemnin B could inhibit the VSVG infection also**.** Among six natural products Scillaren A and Acetoxycyclohexamide showed a moderate level of inhibition (Fig. 8).

### ACE2-S1-binding assay

**Fig. 9:**
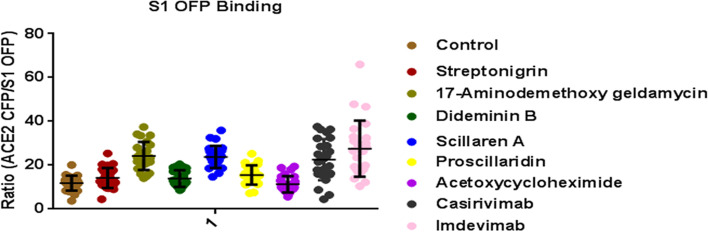
Spike S1 OFP binding study. A higher ratio indicates the magnitude of neutralization. Human monoclonal antibody Casirivimab and Imdevimab showed higher neutralization. 17-Aminodemethoxy geldanamycin and scillaren A showed Spike S1 neutralization. Graph mean ± SD

Further to confirm specificity of the lead compounds to inhibit binding of Spike protein to hACE2, a straight-forward method was employed to image the binding of fluorescent spike protein to hACE2 cerulean. Selected six natural products were incubated with S1-OFP for 1 h and further treated with fixed ACE2 cerulean cells. As shown in Fig. 9, inhibition of S1 protein binding with ACE2 was observed in 17-Aminodemethoxygeldanamycin and Scillaren A treatment when compared with DMSO control. (Fig. 9) suggesting these two compounds possess specific binding inhibition compared to others. Both monoclonal antibodies showed complete inhibition as seen from the increased ACE2/S1OFP ratio substantiating the validity of the method.

### Time-lapse imaging of selected natural products for syncytia formation

**Fig. 10:**
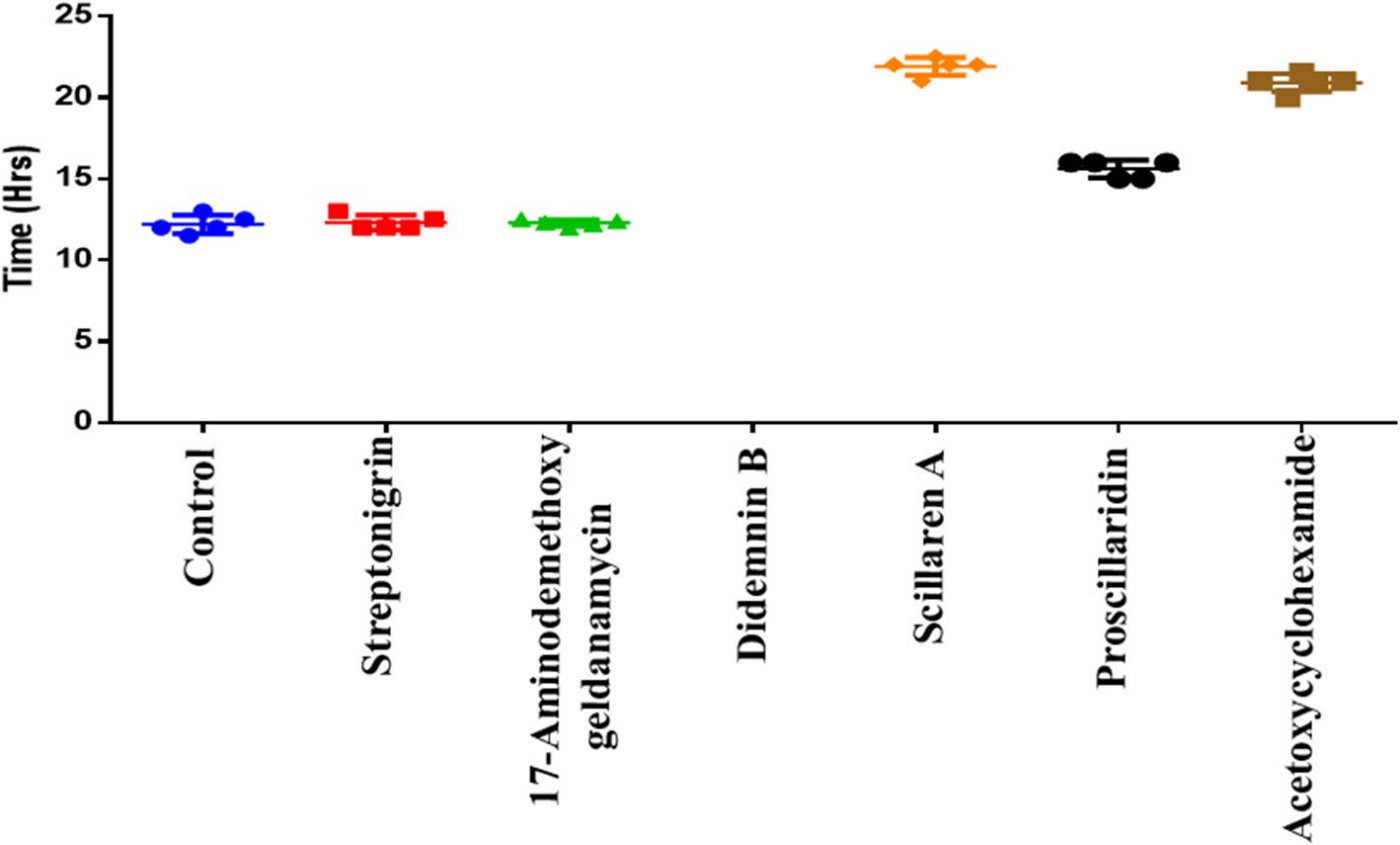
Graph showing initiation of syncytia formation time after VSV-eGFP-SARS-CoV2 infection along with six positive hits in real-time imaging. Data mean ± S.D n = 3 replicate experiments compared to DMSO control

Real-time imaging of pseudovirion infection and syncytia formation was carried out using the lead natural products. The IC_50_ concentration of natural products was used to assess inhibition of syncytia formation. Compared to the control, real-time imaging showed inhibition of pseudovirion infection with natural product treatments. Initiation of pseudoviral infection was observed after 7 hpi in the control, whereas cells treated with natural products Streptonigrin, 17-Aminodemethoxy geldanamycin, Didemnin B, Scillaren A and Proscillaridin showed infection started at 8 hpi and Acetoxycyclohexamide at 10 hpi. ACE2-Spike dependent syncytia formation was initiated in the control after 12 hpi, and no syncytia was observed in Didemnin B treatment upto 24 hpi. Other lead natural products Streptonigrin at 12 hpi, 17 Aminodemethoxy geldanamycin at 12 hpi similar to control. Whereas, other two natural products showed delayed syncytia formation, such as, Scillaren A at 22 hpi, Proscillaridin at 16 hpi and Acetoxycyclohexamide at 21 hpi (Fig. 10 & Supplementary videos 3, 4, 5, 6, 7, 8, 9, 10).

## Discussion

The coronavirus entry mechanism consists of viral Spike glycoprotein binding with host cell receptor ACE-2 and subsequent membrane fusion. Spike protein is a type- I membrane fusion protein consisting of two non-covalently associated subunits S1 (14–685 residues) and S2 (686–1273 residues). S1 binds with ACE2 receptor, whereas S2 subunit secures Spike protein to the membrane and proceeds membrane fusion. The binding of S1 to the ACE2 receptor is the crucial step for SARS-CoV-2 entry. The S1 subunits have four domains such as amino terminal domain, receptor binding domain (RBD) and two carboxy terminal domains. RBD (319–541 residues) consist of a loop termed receptor binding motif (RBM), which binds directly with ACE2. Currently available vaccines are developed based on the Spike protein, which is present in all SARS-CoV-2 variants. However, mutations in the Spike protein enable the SARS-CoV-2 variants to increase its transmissibility, pathogenicity, and minor to moderate antibody escape. Thus, the rapid mutation in Spike protein may necessitate the development of new vaccines and therapeutic antivirals against SARS-CoV-2 [7, 16].

Accelerated targeted drug discovery is crucial in identifying lead molecules against SARS-CoV-2 entry into mammalian cells. Multiple cell-based and cell-free assays have been developed for the identification of modulators of ACE2-Spike binding, and their utility in neutralizing antibody testing [14, 17]. Some of these approaches have also been modified for the discovery of viral entry blockers [6, 18]. However, most cell-based assays are influenced by the toxicity of the host cell, and in general clinically useful viral entry blockers need to be less toxic to mammalian cells. This necessitates sensitive assays for antiviral activity, along with cell toxicity readout. The high-throughput adaptable assay systems described here can precisely identify molecules that can inhibit the binding of SARS-CoV-2 Spike protein with host ACE2 receptors and also toxicity readout along with segmentation possibility utilizing H2B (Fig. 5). H2B phosphorylation occurs later in apoptosis during chromatin condensation and DNA fragmentation making this an ideal real-time sensor [19]. Validation of the assay system with known spike inhibitor Casirivimab, a monoclonal antibody against Spike [20] showed inhibition of VSV-eGFP-SARS-CoV-2 infection without any cytotoxicity, however, dilution-dependent increased infection caused cytotoxicity. The cytotoxic drug, arsenic trioxide induced dose-dependent cytotoxicity, which could be easily detectable through our assay system. In fact, it is observed that infection and cytotoxicity was directly proportional to increasing MOI, indicating virus-induced toxicity. Thus, the developed assay system could report the viral entry inhibition and cytotoxicity of the host cells (Fig. 4). For the screening of large compound libraries, H2B mCherry intensity with pseudovirion infection in the scatter plot represents the severity of the infection using high throughput imagers as well as with conventional low-cost fluorescent microscopy setting as described. The inhibition of pseudo viral infection readout may be influenced by the toxicity of natural products. If pseudo viral infection was the only evaluation parameter, high-throughput screening may identify this as a false positive hit [10]. However, the present reporter assay system, which integrates H2B mCherry, enables to ascertain viability status of cells also (Fig. 4).

The replication-competent VSV-eGFP-SARS-CoV-2 pseudovirion assay system, which is compatible with biosafety level 2 (BSL-2), performs similarly to the clinical isolate of SARS-CoV-2 [12] and enables the detection of syncytia formation. In our primary screening, we identified 24 natural products as primary hits with > 80% inhibition of infection at a concentration of 10 µM (Fig. 6A, B). The primary and secondary screening was mainly focused on the infection – inhibition based on the VSV-eGFP-SARS-CoV-2 pseudovirion assay. Among the 24 positive hits, six were further identified as leads based on the toxicity and ability to inhibit infection even at a higher MOI (0.8). Specifically, natural products 17-Amino dimethoxy geldanamycin, Didemnin B, Streptonigrin, Scillaren A, Proscillaridin, and Acetoxycycloheximide exhibited SARS-CoV-2 entry inhibition at a concentration of 1 µM (Fig. 6 and 7, Supplementary Fig. 4). 17-Amino dimethoxy geldanamycin is a geldanamycin analogue that inhibits heat shock protein-90 (HSP-90). Recent studies showed, HSP90 inhibitor SNX-5422 significantly inhibited SARS-CoV-2 replication and could be used as an early therapy and potentially reduce disease severity, improve clinical conditions, decrease hospitalization of COVID-19 patients [21].

VSV inhibition also could reduce the GFP expression and necessitates additional secondary assays for ensuring specificity such as direct binding assays using recombinant proteins or VSVG approach as described. Similarly, preincubation of 17-Amino dimethoxy geldanamycin with host cells showed a moderate level of viral entry inhibition indicating its ability to modulate host ACE2. However, 17-Amino dimethoxy geldanamycin could remarkably inhibit Spike binding (S1) and pseudovirion entry (Fig. 9 and Supplementary Fig. 6). Thus 17-Amino dimethoxy geldanamycin may inhibit viral entry through host cell modification and spike binding, further studies are necessary to explore the mechanism of action. Didemnin B cyclic depsipeptide marine natural product with antiviral properties has been reported to inhibit the main protease (MPro) of COVID-19 [22]. Didemnin B-derivative plitidepsin (Aplidine) or dehyrodidemnin B has been reported to have potent SARS-CoV-2 inhibition by targeting host eukaryotic translation elongation factor 1A [23]. Our studies also showed preincubation of Didemnin B with host cells could inhibit viral infection, this may be to the host cell response against viral entry (Supplementary Fig. 5). Streptonigrin tetracyclic aminoquinoline-5,8 dione is an antitumor antibiotic, and previous screening against SARS-CoV-2 nsp15 endoribonuclease identified it as a positive hit [24]. Scillaren A is a cardiac glycoside derived from Drimia species [25] and in silico studies have been reported to have SARS-CoV-2 Mpro binding affinities [26]. In our present study, Scillaren A inhibits pseudovirion infection and Spike S1 binding. Proscillaridin is a cardiac glycoside isolated from *Drimia maritima*, an anticancer drug that inhibits DNA topoisomerase I and II [27]. Acetoxycycloheximide is an acetylated derivative of cycloheximide, which inhibits translation elongation by binding to the ribosomal E-site [28].

The lead compounds induced a moderate level of cytotoxicity along with pseudovirion inhibition with an exception in streptonigrin that showed higher cytotoxicity. H2B nuclear condensation and Dose–response studies with or without VSV-eGFP-SARS-CoV-2 pseudovirion showed natural products having minimum toxicity levels at IC_50_ concentration (Fig. 7 and Supplementary Fig. 4).

The formation of syncytia, which is mediated by the spike protein, increases the spread of the virus and the complexity of the disease. Therefore, there is a need to develop assay systems and conduct drug screening that inhibits syncytia formation [14, 17]. Notably, the spike protein alone can trigger syncytia formation in SARS-permissive cells without the presence of any other viral protein [14]. In our present study, syncytia formation was observed 12 h after infection, which is consistent with a previous observation [29]. The formation of syncytia, generated by the VSV-eGFP-SARS-CoV-2 pseudovirion in SiHA ACE2 cerulean H2B mCherry cells, proves to be a promising imaging tool for studying the fusion mechanism of the Severe acute respiratory syndrome (SARS) virus and subsequent viral entry in real-time.

Further studies are needed to assess the efficacy of the lead natural products against clinical isolates of SARS-CoV-2. Our present approach, which simultaneously screens for pseudoviral inhibition, toxicity, and ACE2-Spike mediated syncytia formation, offers multiple advantages. To validate the cell system, we attempted the screening using only a small number of natural products that led to the identification of a few leads. Interestingly, most of the leads were previously reported to have antiviral activity, confirming the sensitivity of the assay system. Since the cell system also supports real-time imaging of syncytia formation and cytopathic changes without fixation or pre-processing, it will be an ideal tool for antiviral discovery using live virus.

## Conclusion

The developed robust BSL-2 compatible assay system is suitable for high-throughput screening of viral entry inhibitors against SARS-CoV-2 along with toxicity analysis and effect on syncytia formation. The model system described is a promising tool to screen large compound libraries equivalent to the infectious SARS-CoV-2 and also support live virus-based assays.

## Supplementary Information

**Additional file 1: Supplementary Video 1. **Validation of SARS CoV2 permissive reporter system with pseudovirion infection**. **Live cell imaging showing VSV-eGFP-SARS-CoV2 infection in SiHa ACE2 Cerulean H2B mCherry cells.

**Additional file 2: Supplementary Video 2. **Validation of SARS CoV2 permissive reporter system for syncytia formation with VSV-eGFP-SARS-CoV2 infection.Live cell imaging showing Syncytia formation in SiHa ACE2 Cerulean H2B mCherry cells, 12 hours post-infection of Pseudovirion. Imaging were started 1hpi and continued upto 33 hpi.

**Additional file 3: Supplementary Video 3.** Time-lapse imaging of SiHa ACE2 Cerulean H2B mCherry cells without VSV-eGFP-SARS-CoV2 infection showing no syncytia formation. Imaging was started at 4 hpi and continued up to 24 hpi.

**Additional file 4: Supplementary Video 4. **Time-lapse imaging of control cell showing syncytia formation. Imaging was started at 4 hpi and continued up to 24 hpi.

**Additional file 5: Supplementary Video 5. **Time-lapse imaging of evaluation of syncytia formation after Streptonigrin treatment. Imaging was started at 4 hpi and continued up to 24 hpi.

**Additional file 6: Supplementary Video 6. **Time-lapse imaging of evaluation of syncytia formation after 17- Amino dimethoxy geldanamycin treatment. Imaging was started at 4 hpi and continued up to 24 hpi.

**Additional file 7: Supplementary Video 7. **Time-lapse imaging of evaluation of syncytia formation after Didemnin B treatment. Didemin B inhibits syncytia formation**. **Imaging was started at 4 hpi and continued up to 24 hpi.

**Additional file 8: Supplementary Video 8. **Time-lapse imaging of evaluation of syncytia formation after Scillaren A treatment. Imaging was started at 4 hpi and continued up to 24 hpi.

**Additional file 9: Supplementary Video 9. **Time-lapse imaging of evaluation of syncytia formation after Proscillaridin treatment. Imaging was started at 4 hpi and continued up to 24 hpi.

**Additional file 10: Supplementary Video 10. **Time-lapse imaging of evaluation of syncytia formation after Acetoxycyclohexamide treatment. Imaging was started at 4 hpi and continued up to 24 hpi.

**Additional file 11: Supplementary Fig 1. **Flow cytometry scatter plot of SiHa cells as Negative control.

**Additional file 12: Supplementary Fig. 2. (A.) **Western blot of TMPRSS2 protein in SiHa, SiHa ACE2 Ceruelan H2B mCherry and MDAMB 468 cell line. (**B.)** FACS analysis showing presence of TMPRSS2 in SiHa cells (Pink) compared to the unstained control (Blue).

**Additional file 13: Supplementary Fig. 3. **Validation of assay system with different MOI of pseudovirion, A). Microscopic images of different MOI (0.7 to 0.01) images were captured under 20x objective with 0.75 NA after 12 hpi. B). Graphshowing the effect of different MOI titer on pseudovirion infection and cellular toxicity.

**Additional file 14: Supplementary Fig. 4. **Graph showing H2B Nuclear condensation of Six natural products (µM) alone in SiHa ACE2 Cerulean H2B mCherry Cells.  Data mean ±S.D n=3 replicate experiments compared to DMSO control.

**Additional file 15: Supplementary Fig. 5. **Graph showing VSV-eGFP-SARS-CoV2 inhibition A. 1 hr infection of VSV-eGFP-SARS-CoV2 and Natural product (1 µM) together in SiHa Ace2 cerulean H2B mCherry cells. B. 1 hr preincubation of SiHa Ace2 cerulean H2B mCherry cells with Natural product (1 µM) and further 1 hr VSV-eGFP-SARS-CoV2 infection. Data mean±S.D n=3 replicate experiments compared to DMSO control.

**Additional file 16: Supplementary Fig. 6. **Spike S1 OFP binding study. A higher ratio indicates the magnitude of neutralization. Human monoclonal antibodies Casirivimab and Imdevimab showed higher neutralization. 17-Aminodemethoxy geldanamycin and scillaren A showed Spike S1 neutralization. Graph mean± SD

**Additional file 17: Supplementary fig 7.**

## Data Availability

All data supporting the results and conclusion in the manuscript are present in the manuscript and/or the Supplementary Materials. Additional data related to this paper may be requested from the authors. Cell resources and plasmids generated in this study are available upon request after MTA agreement.
