# Supplementary figures and images for: A high-throughput screening system for SARS-CoV-2 entry inhibition, syncytia formation and cell toxicity
Source: Biol Proced Online. 2023 Jul 26;25:22. doi: 10.1186/s12575-023-00214-1 (PMC10373420; doi:10.1186/s12575-023-00214-1)

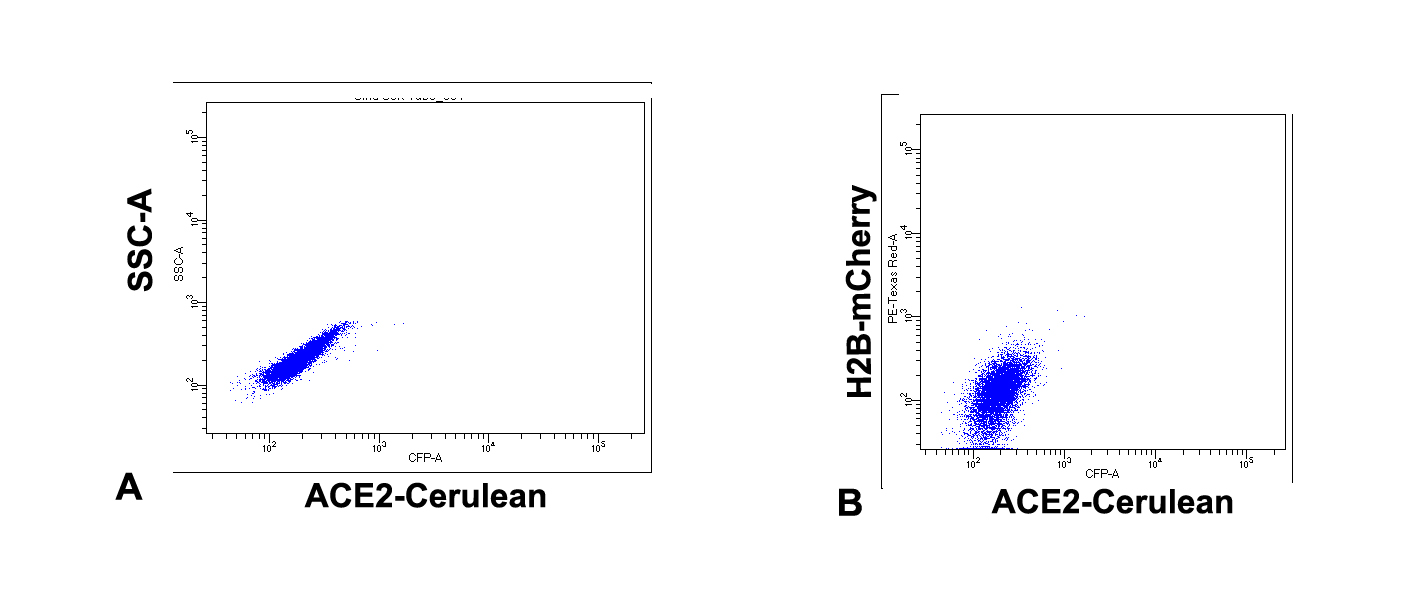

Supplement: Supplementary file 11 — Additional file 11: Supplementary Fig 1. Flow cytometry scatter plot of SiHa cells as Negative control. [file 12575_2023_214_MOESM11_ESM.jpg]

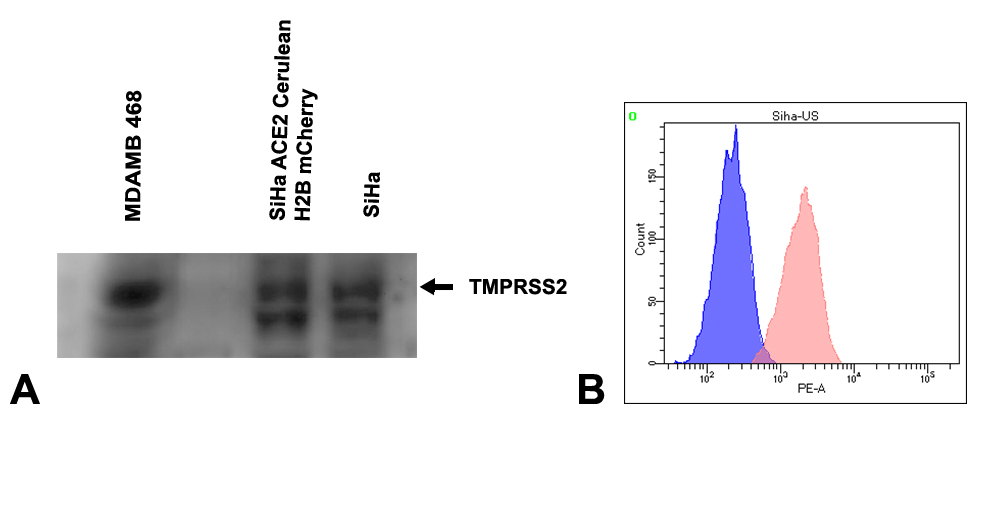

Supplement: Supplementary file 12 — Additional file 12: Supplementary Fig. 2. (A.) Western blot of TMPRSS2 protein in SiHa, SiHa ACE2 Ceruelan H2B mCherry and MDAMB 468 cell line. (B.) FACS analysis showing presence of TMPRSS2 in SiHa cells (Pink) compared to the unstained control (Blue). [file 12575_2023_214_MOESM12_ESM.jpg]

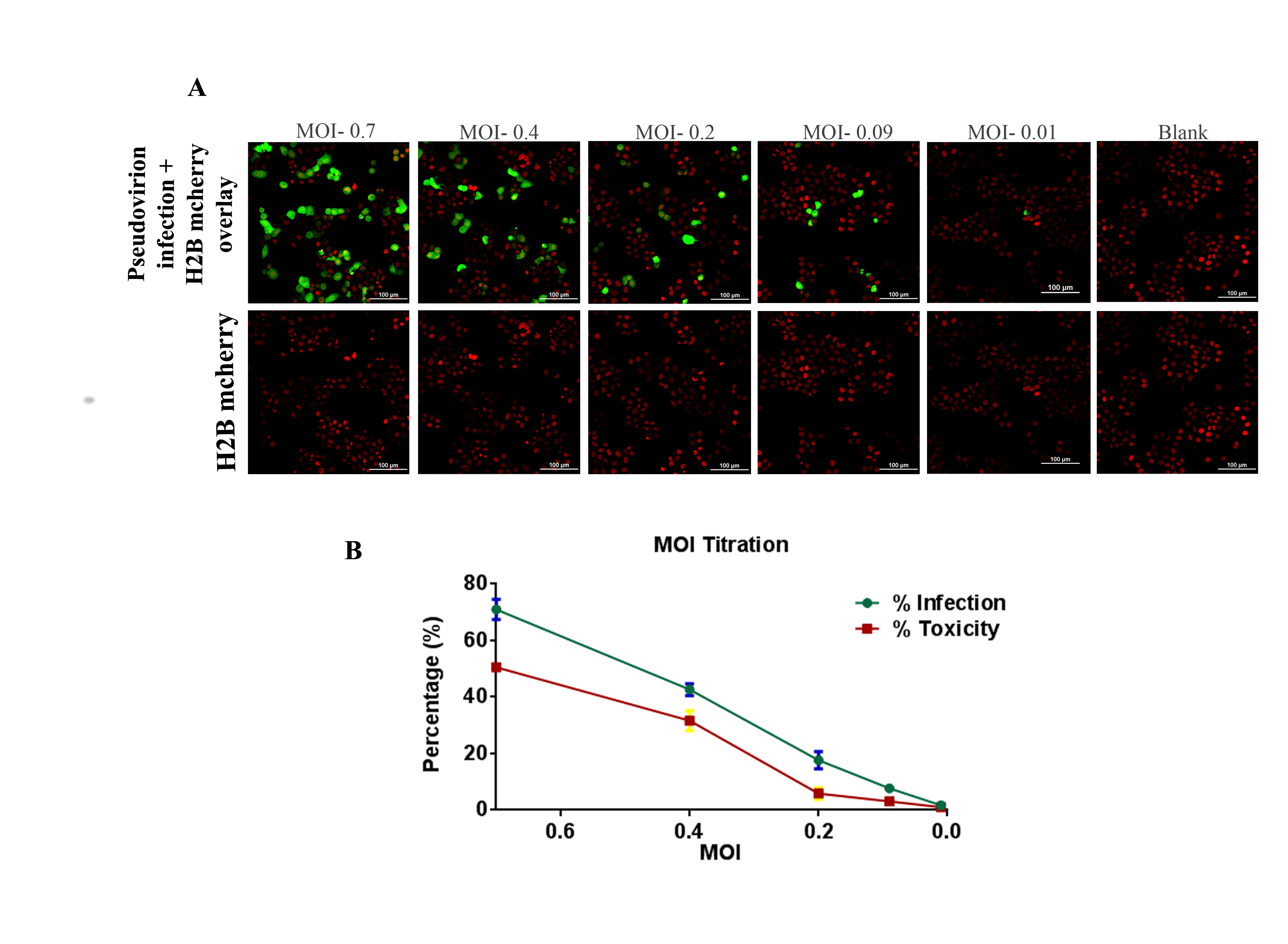

Supplement: Supplementary file 13 — Additional file 13: Supplementary Fig. 3. Validation of assay system with different MOI of pseudovirion, A). Microscopic images of different MOI (0.7 to 0.01) images were captured under 20x objective with 0.75 NA after 12 hpi. B). Graphshowing the effect of different MOI titer on pseudovirion infection and cellular toxicity. [file 12575_2023_214_MOESM13_ESM.jpg]

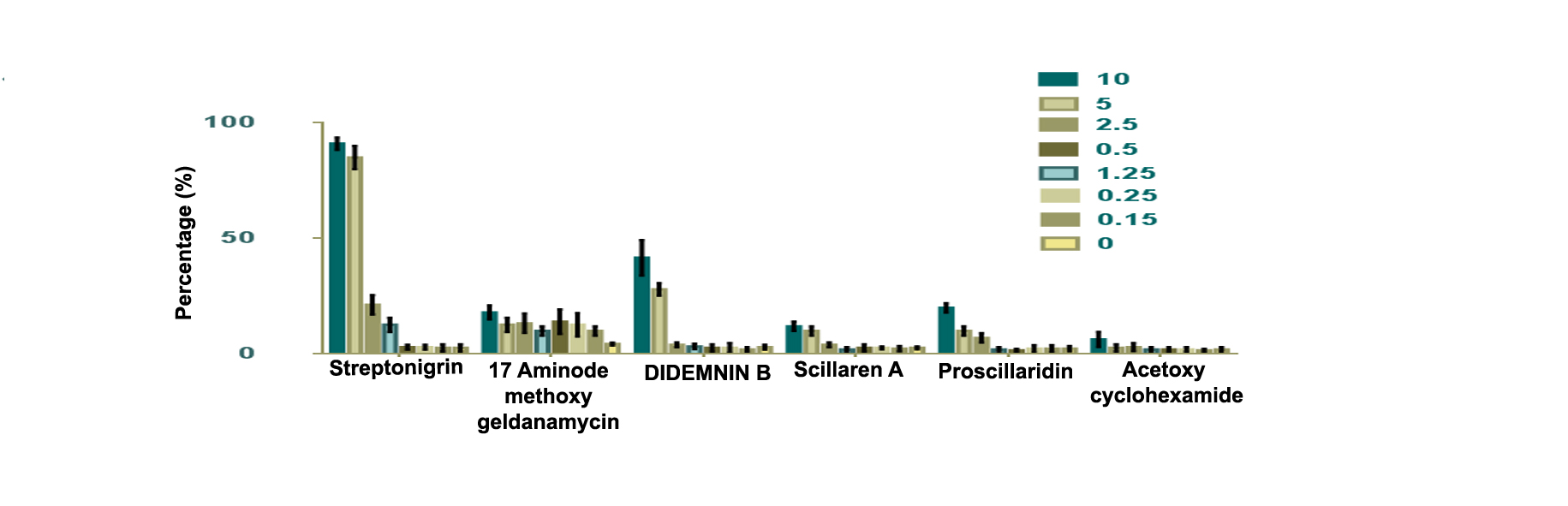

Supplement: Supplementary file 14 — Additional file 14: Supplementary Fig. 4. Graph showing H2B Nuclear condensation of Six natural products (µM) alone in SiHa ACE2 Cerulean H2B mCherry Cells. Data mean ±S.D n=3 replicate experiments compared to DMSO control. [file 12575_2023_214_MOESM14_ESM.jpg]

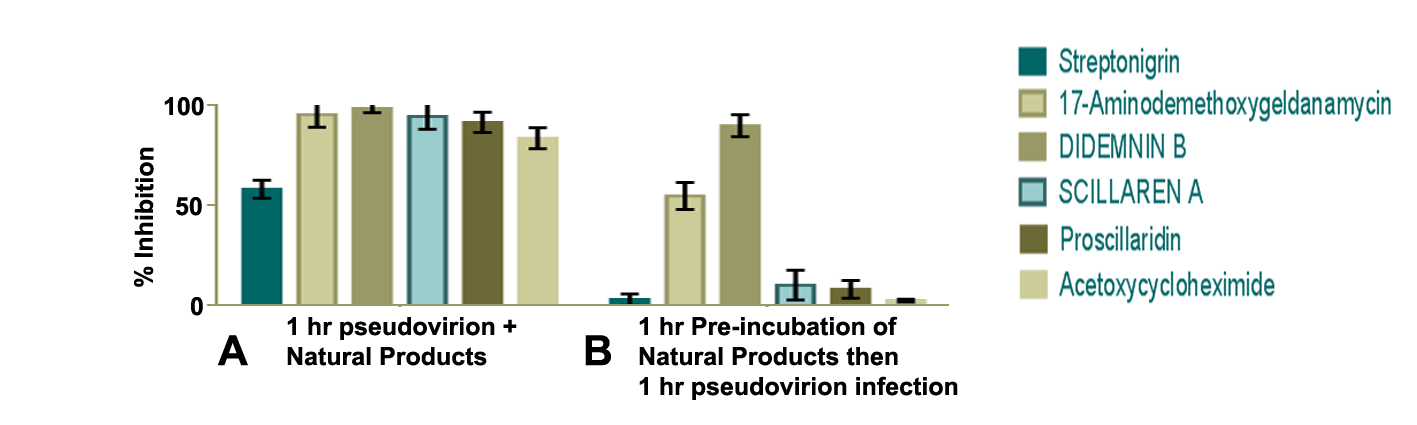

Supplement: Supplementary file 15 — Additional file 15: Supplementary Fig. 5. Graph showing VSV-eGFP-SARS-CoV2 inhibition A. 1 hr infection of VSV-eGFP-SARS-CoV2 and Natural product (1 µM) together in SiHa Ace2 cerulean H2B mCherry cells. B. 1 hr preincubation of SiHa Ace2 cerulean H2B mCherry cells with Natural product (1 µM) and further 1 hr VSV-eGFP-SARS-CoV2 infection. Data mean±S.D n=3 replicate experiments compared to DMSO control. [file 12575_2023_214_MOESM15_ESM.jpg]

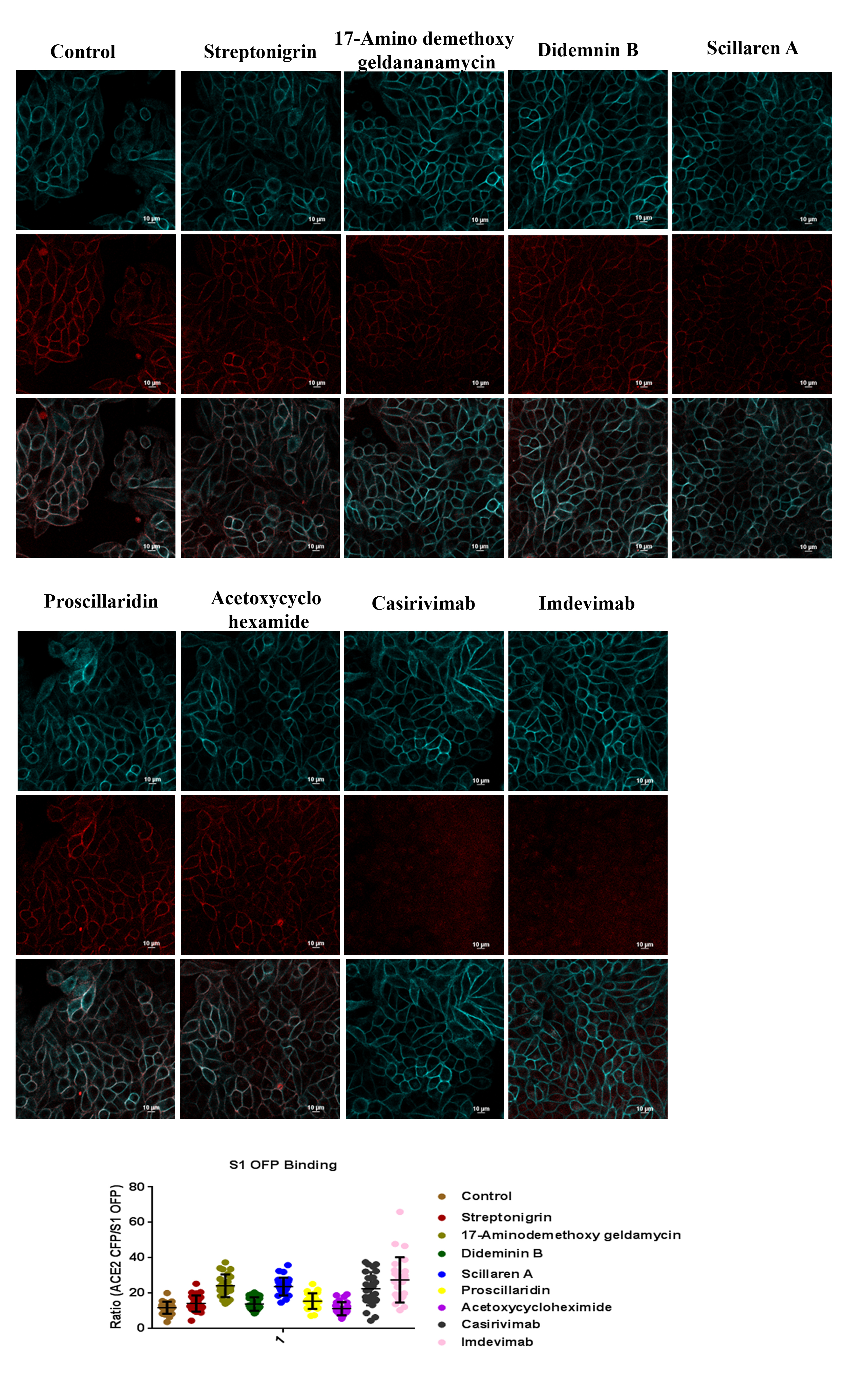

Supplement: Supplementary file 16 — Additional file 16: Supplementary Fig. 6. Spike S1 OFP binding study. A higher ratio indicates the magnitude of neutralization. Human monoclonal antibodies Casirivimab and Imdevimab showed higher neutralization. 17-Aminodemethoxy geldanamycin and scillaren A showed Spike S1 neutralization. Graph mean± SD [file 12575_2023_214_MOESM16_ESM.jpg]

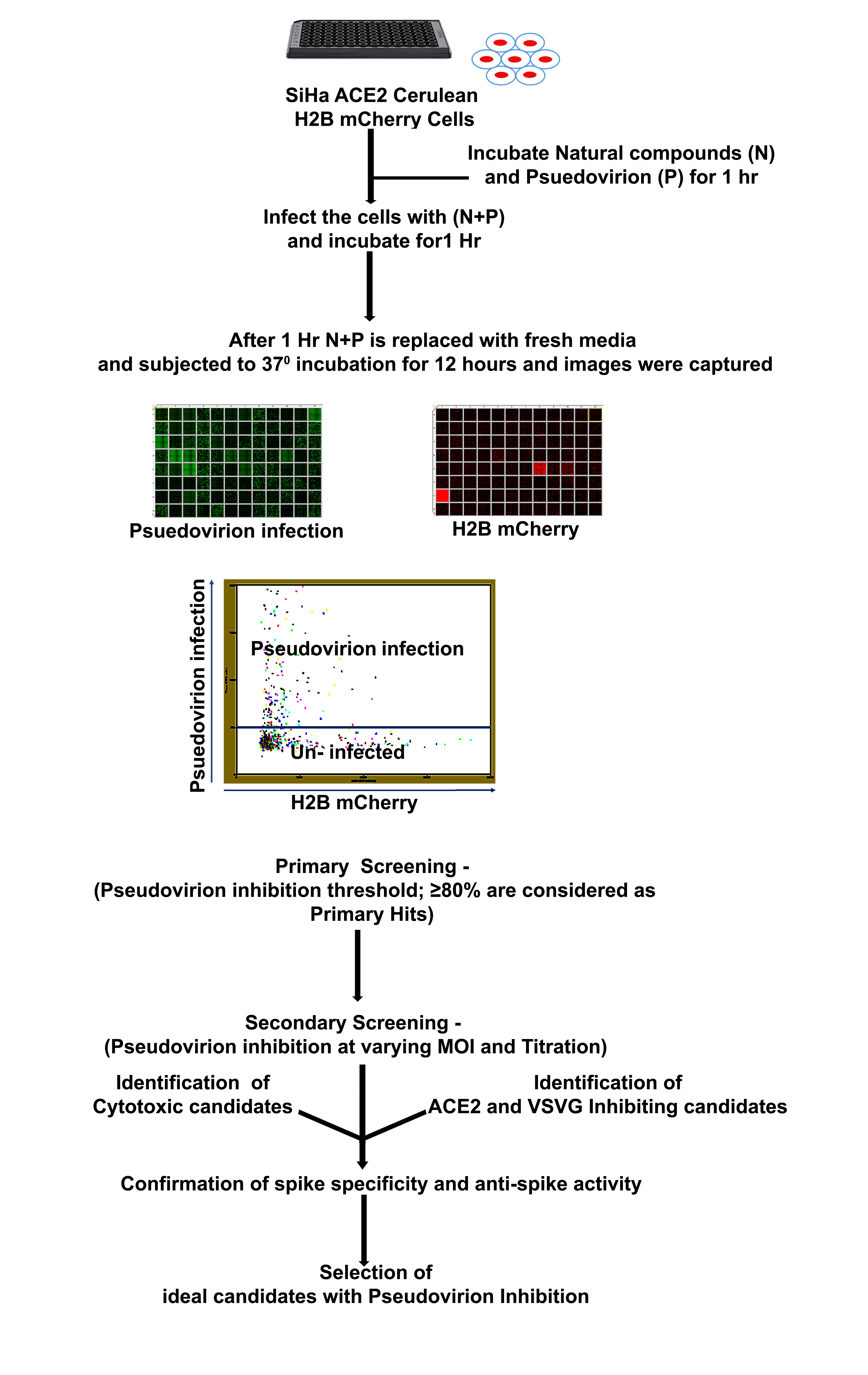

Supplement: Supplementary file 17 — Additional file 17: Supplementary fig 7. [file 12575_2023_214_MOESM17_ESM.jpg]
